# Quality Related Safety Evaluation of a South African Traditional Formulation (PHELA^®^) as Novel Anti-Biofilm Candidate

**DOI:** 10.3390/molecules27041219

**Published:** 2022-02-11

**Authors:** Bhaskar Das, Amit Kar, Rudranil Bhowmik, Sanmoy Karmakar, Satyajit Tripathy, Motlalepula G. Matsabisa, Pulok Kumar Mukherjee

**Affiliations:** 1 Department of Pharmaceutical Technology, School of Natural Product Studies, Jadavpur University, Kolkata 700032, India; mailmebhaskar007@gmail.com (B.D.); rudranilz@yahoo.in (R.B.); sanmoykarmakar@gmail.com (S.K.); naturalproductm@gmail.com (P.K.M.); 2 Institute of Bioresources and Sustainable Development, Department of Biotechnology, Government of India, Imphal 795001, India; amit.kar2@gmail.com; 3 Department of Pharmacology, School of Medicine, Faculty of Health Sciences, University of the Free State, Bloemfontein 9300, South Africa; TripathyS@ufs.ac.za

**Keywords:** PHELA^®^, South African traditional preparation, RP-HPLC, herb-drug interaction, antibacterial, biofilm

## Abstract

A South African traditional formulation, PHELA^®^, is consumed by the traditional people for severe chest problems with coughing, diarrhea, oral ulcers etc. The present study focused on establishing the anti-infective properties of a safe and standardized poly-herbal formulation through a series of criteria and specifications.

## 1. Introduction

Biofilms are aggregates of microorganisms entrenched in a self-produced extracellular polymeric substances (EPS) matrix by adhering to each other and/or abiotic surface having high cell densities, ranging from 10^8^ to 10^11^ cells/gm wet weight [1,2,3,4]. Around 60% of human infections are due to formation of biofilm. Most of the biomass of the biofilms is made up of hydrated EPS rather than microbial cells. The spatial organization of EPS molecules in the matrix is a nonstop, dynamic process that produces microcolonies from the biofilm cluster [5]. The functional and emergent property of the matrix is due to the heterogeneous nature of the polysaccharides in a highly ordered composition. Biofilms allow microorganisms to trap nutrients and withstand hostile environmental conditions (pH, temperature and presence of antibiotics) through quorum sensing (QS) [6]. QS helps the biofilms to respond to the environmental changes and facilitate the colonization of a surface by forming streamers. This increases antimicrobial resistance compared to free-living bacterial cells. Due to these emergent properties, it is challenging to target biofilm forming microorganisms in industrial and health care sectors. Natural products have gained extensive interest in the search of alternatives for anti-microbial therapy [7]. The compounds are well accepted due to safety reasons and having a long history of use in different traditional medicine for the prevention and treatment of infectious diseases [8,9,10]. The use of herbal medicines has significantly increased over last decades. In comparison with a single herb, herbal combinations have shown a more promising effect in the treatment of disease conditions. The concept of poly herbal combinations has been well established and unexpected success has been reached. However, traditional herbal treatments are not found to be inherently safe due to their unexpected interactions with different drug metabolizing enzymes when prescribed with conventional medicines. A survey states that about 14–31% of prescription drug users take the herbal products as a nutritional supplement. The lack of adequate knowledge of interaction potential with these herbs with drug metabolizing enzymes results in altered pharmacokinetic and/or pharmacodynamic parameters. More than 70% of drug candidates are being bio-transformed by Cytochrome P450 class of isozymes via oxidation. Thus, the documentation of the herbal medicine interacting with CYP isozymes became essential for regulatory licensing using different in-vitro, in-vivo and in-silico techniques [11].

PHELA^®^ is a crude polyherbal formulation, used in the South African traditional system of medicine, considered as a dietary supplement that has a traditional use as “Imbiza” for blood cleansing. Different parts of the four plants, namely, *Gladiolus dalenii* Van Geel, *Clerodendrum glabrum* E. Mey, *Senna occidentalis* (L.) Link and *Rotheca myriciodes* (Hochst), were combined by Steane & Mabb in a specific ratio to make the PHELA^®^ formulation. The herbal formulation has been reported to have benefits for the condition traditionally known as “Muyaka”, characterized in bed-ridden patients with severe chest problems, coughing, coated or pimply tongue, resulting in severe weight and appetite loss, vomiting and diarrhea, stiffness, oral ulcers and slow painful death [12,13]. Few studies are available reporting on immune stimulation and anti-Alzheimer’s potentials of the formulation [14,15]. The phytochemical composition of the formulation has yet not been documented well. A comprehensive chromatographic method was developed by Lekhooa et al., for fingerprinting analysis of different solvent extracts [16]. In another study, the developed HPLC-FLD analysis identified major peaks in rat plasma after administration of aqueous extract at different time intervals. The developed method could help to optimize the pharmacokinetic parameters to obtain steady state plasma concentration [17].

The present study is undertaken to investigate the biofilm inhibitory property of standardized PHELA^®^ extract and to evaluate its interaction potential with cytochrome class of drug metabolizing enzymes.

## 2. Materials and Methods

Metabolite profiling was performed by UPLC-MS/MS followed by quantitative estimation of major metabolite by RP-HPLC analysis. The cytotoxicity assay was studied in HEP G2 cell line, and interaction potential with different drug metabolizing enzymes was performed using CYP450 isozymes inhibition assay method. The antibacterial potency of the extracts was studied against *Staphylococcus aureus*, *Bacillus substilis*, *Escherichia coli*, *Pseudomonas aeruginosa*. Standard plate count method followed to understand the effect of the extract on the bacterial growth pattern and reported as CFU/mL. Inhibition of biofilm formation study was performed using crystal violet stain and AFM was utilized to unravel its surface topology.

### 2.1. Chemicals and Reagents

LC grade methanol (Merck-Mumbai, India), emodin (>98%), ketoconazole and fluoxetine from Sigma chemicals (Steinheim, Germany). Vivid^®^ CYP450 Screening Kit [CYP2D6 (Cat. No. P2972) and CYP3A4 (Cat. No. P2858)] and specific Vivid^®^ Substrates [CYP2D6—EOMCC; CYP3A4—BOMCC] were purchased from Life Technologies (Carlsbad, CA, USA), Muller Hinton Broth (MHB), Nutrient Agar and Crystal violet were supplied by Himedia, India. All other chemicals and reagents were of analytical grade (E-Merck). Bacterial strains like *Staphylococcus aureus* (ATCC 29213), *Bacillus cereus* (ATCC 14579), *Pseudomonas aerugenosa* (ATCC 9027) and *Escherichia coli* (ATCC 25922) were sub-cultured on Mueller Hinton Broth (MHB) and incubated at 37 °C for 24 h.

HEPG2 cells were obtained from the American Type Culture Collection (ATCC HB 8065). Eagle’s Minimum Essential Media (EMEM), Fetal Bovine Serum (FBS) and Phosphate Buffer Saline (PBS) were purchased from Life technologies Ltd. (Fairlands, Johannesburg, South Africa). The 3-(4,5-Dimethylthiazol-2-yl)-2,5-Diphenyltetrazolium Bromide (MTT), Dimethyl Sulfoxide (DMSO), Trypsin and all other chemicals and reagents were of analytical grade and acquired from Merck (Pvt.) Ltd. (Modderfontein, Johannesburg, South Africa).

The cell line was maintained in EMEM supplemented with 10% FBS, supplemented with non-essential amino acids and sodium pyruvate (1 mM). The cell line was grown at 37 °C in a humidified incubator set at 5% CO_2_ and sub-cultured with 0.25% (*w*/*v*) trypsin and 0.53 mM EDTA for a maximum of 15 min every 2–3 days after they had formed a 90% confluent monolayer.

### 2.2. Procurement of Plant Material

Crude PHELA^®^ sample was provided by Prof. Motlalepula G. Matsabisa, the Director, IKS Lead Programme, Department of Pharmacology, University of Free State, Bloemfontein, South Africa under the India–South African Science and Technology funded bilateral research project (DST-NRF Unique Grant No. 104775 UFS Entity 1 047 N1418). The voucher specimens for individual plant *Gladiolus dalenii* Van Geel (BLFU/MGM001), *Clerodendrum glabrum* E. Mey (BLFU/MGM004), *Senna occidentalis* (L.) Link (BLFU/MGM0012) and *Rotheca myriciodes* (Hochst), Steane & Mabb (BLFU/MGM0013) were stored at Department of Pharmacology, Faculty of Health Sciences, University of the Free State, Bloemfontein, South Africa. For PHELA^®^ formulation, the crude herbal formulation was ground into coarse powder and mixed (1:4:8:6).

### 2.3. Extraction of the Plant Material

The crude powdered PHELA^®^ formulation was subjected to hydro-alcoholic extraction through maceration. 100 g of the milled plant sample was soaked in 500 mL of 80% methanol at room temperature with frequent shaking for 72 h. The extraction procedure was repeated 3 times. Finally, the filtrates were pooled and concentrated using a rotary vacuum evaporator (IKA, Osaka, Japan) and lyophilized (Instrumentation India, India) to get dry powder. The percentage yield for the hydro-alcoholic extract of PHELA^®^ (PHAE) was found to be 13.5% (*w*/*w*). The dried extract was packed in an airtight container and stored at 4 °C freezer for further use.

### 2.4. UPLC-MS Analysis of PHELA^®^ Extract

Compound separation and detection was performed using a Waters UPLC hyphenated with a Waters Synapt G2 QTOF instrument. Analysis was done in an Acquity UPLC BEH C 18 1.7 µm (2.1 × 100 mm column). The column was operating in a flow rate of 0.4 mL/min. For negative ion mode the mobile phase used was A; 0.1% HCO_2_H in deionized water and B; MeOH + 0.1% HCO_2_H. MS source ESI operated in positive, capillary voltage and endplate voltage were set at 4000 V and −500 V, respectively. Nitrogen was used as nebulizing gas at 35 p.s.i. and *m*/*z* range was set from 120 to 1000 amu and for positive ion mode the mobile phase consisted of A; 5 mM ammonium formate buffer (pH 8) contained 1% methanol, and solvent (C) 100% methanol. Gradient elution started with 95% A and 5% B at 40 °C which remained at the linear gradient until 15 min. From 15 to 17 min it was 10% A and 90% B, then, from 17 min to 21 min, elution was kept constant into 0% A and 100% B, a linear gradient was then used to reach completion, 95% A and 5% B until 25 min.

### 2.5. RP-HPLC Analysis of PHELA^®^ Extract

The percentage content of standard phytomarker emodin in PHELA^®^ extract was determined through RP-HPLC (Shimadzu Prominence, Kyoto, Japan) system. Built with two Shimadzu LC-20 AD UFLC reciprocating pumps, a variable Shimadzu SPD-M20A Prominence PDA detector and a Rheodyne manual injector with a loop volume of 20 μL with a C18column (Phenomenex-Luna C18, Torrance, CA, USA) (250 × 4.6 nm, 5 μm particle size) was used as the stationary phase. For analysis, the standard and test samples were dissolved in methanol to obtain a stock solution of 1 mg/mL. The standard and sample were injected at a volume of 20 μL using Hamilton Micro liters syringe (Bonaduz, Switzerland). The analysis was performed under isocratic elution using optimized mobile phase methanol and 1% acetic acid solution at a 1 mL/min flow rate. The detection of the compounds was performed at λ_max_254 nm. The amount of the standard present in the sample was determined through the construction of the calibration curve by plotting peak area against corresponding concentrations through linear regression using LC solution software [18].

### 2.6. Safety Assessment of PHELA^®^ Extract

#### 2.6.1. MTT Cytotoxicity Assay

The toxicity of PHELA^®^ extract in HEPG2 cells were performed using MTT cytotoxicity assay with some modification as described by Kar et al. [19]. HEPG2 cells were cultured and maintained in high glucose Dulbecco’s modified Eagle’s medium (DMEM) supplemented with 10% (*v*/*v*) fetal bovine serum, L-glutamine (4 mM), 100 U/mL penicillin and 100 µg/mL streptomycin. Cells (1 × 10^4^ cells/mL) were seeded in 100 µL medium in 96-well microtiter plates and incubated for 24 h at 37 °C and 5% CO_2_ to allow cells to adhere. Subsequently, cells were exposed to the different concentrations of the samples and the controls, which included vehicle-treated cells exposed to 0.5% DMSO; cells propagated in growth medium and cells exposed to the positive control. After the 24 h of treatment period, the cells were exposed to the MTT reagent (0.5 mg/mL). The colorimetric reaction was measured by means of a plate reader (Multiskan Go, Thermofischer Scientific, Vantaa, Finland) at 570 nm. Color control blanks were included and utilized to normalize the results and the vehicle control treated cells were regarded as 100% cell viability. The results given are representative of the average percentage inhibition of all the experiments were repeated as triplicate and given as CC_50_.

#### 2.6.2. CYP450 Enzyme Inhibition Study

Fluorogenic assay was performed in black 96-well microplates. All the samples and standard inhibitors were dissolved in 100 mM Vivid CYP450 reaction buffer to obtain desired concentrations. The assay mixture consisted of 50 μL of prepared enzyme mixture and 40 μL test solution; incubate for 20 min at 37 °C. The enzymatic reaction was initiated by the addition of a mixture of NADP+ and the appropriate substrate (10 μL) and incubated for 10 min at 37 °C. To start the reaction 10μM concentration of 7-benzyloxymethyloxy-3-cyanocoumarin (BOMCC) and 7-ethoxymethoxy-3-cyanocoumarin (EOMCC) fluorogenic substrate were used for CYP3A4 and CYP2D6, respectively. Product formation from the fluorogenic substrate were measured on a fluorescence microplate reader (PHERAstar, BMG Labtech, Cary, NC, USA) using an excitation wavelength of 415 nm and emission wavelength of 460 nm. Percentage inhibition and IC_50_ value were determined [19,20].

### 2.7. Antibacterial and Anti-Biofilm Activity of PHELA^®^ Extract

#### 2.7.1. MIC Determination by Broth Dilution Method Using the 96-Well Plate

Broth dilution is one of the most basic antimicrobial susceptibility testing methods to determine MIC using a 96-well microtitre plate [21]. To each well 50 µL of MHB was dispensed followed by addition of 100 µL of test extract or standard antimicrobial agent (ciprofloxacin, 1 mg/mL) and serially diluted row-wise. Then, each well was inoculated aseptically with 50 µL of bacterial inoculums adjusted to 0.5 McFarland Standard (1.5 × 10^8^ CFU/mL). The well without extract or standard antimicrobial agent served as negative control. Wells containing the extract or standard antimicrobial agent served as test and positive control groups, respectively. Finally, 30 µL of 0.02% p-iodonitrotetrazolium chloride (INT) was added to each well and incubated at 37 °C for 24 h. The MIC concentration determined by change in color.

#### 2.7.2. Determination of MBC

MBC is the lowest concentration of the test material to diminish 99.9% of viable bacterial growth. To determine MBC the contents of MIC wells sub-culturing on agar medium without disinfectant. After 16–20 h of incubation at 35 °C, the number of colonies were counted by colony counter. The experiments were conducted in triplicate to minimize errors [22].

#### 2.7.3. Growth Curves Analysis

As the tested plant extract showed the lowest MIC value against *S. aureus*, the microorganism was cultured on MHB and the bacterial cell concentration was adjusted to 0.5 McFarland standards. Then, 1 mL of the sample mixture was withdrawn at different time intervals (0, 5, 10 and 20 h). At each time interval, the withdrawn samples were serially diluted as 10, 100, 1000 times and spread on agar plates by spread plate technique. The standard plate count method was performed after 24 h of incubation at 37 °C to detect the number of viable bacterial cells in terms of colony forming unit (CFU) for the control and treated isolates using the standard protocol and reported as CFU/mL [23].

#### 2.7.4. Inhibition of Biofilm Formation

The study was performed as described by Sarkar et al. [19]. In a 96=well microtitre plate, 25 μL of a 1.5 × 10^8^ CFU/mL suspension of *S. aureus* was inoculated in 100 µL of fresh MHB in each well followed by the addition of 50 µL of the extract (MIC, MIC X 2, MIC X 3 and MIC X 4) or standard antimicrobial agent (ciprofloxacin) at a sub-lethal concentration and serially diluted row-wise. The well without extract or standard antimicrobial agent served as non-treated control and wells containing the extract or standard antimicrobial agent served as test and positive control groups, respectively. After 16 h of incubation at 37 °C, the plate was taken out and washed with PBS (pH 7.4) to remove non-adherent bacteria. The adherent cells were stained with 1% crystal violet solution followed by washing again with PBS to remove the excess stain. The biofilm was extracted with 33% glacial acetic acid and the absorbance was recorded at 492 nm (SpectramaxM5; Molecular Devices, San Jose, CA, USA) [24].

The percentage of inhibition of biofilm formation was calculated as follows:

(1) $$\frac{\left(1-OD\;of\;the\;test\right)}{\left(OD\;of\;negative\;control\right)}\times 100$$

#### 2.7.5. AFM Assisted Characterization of the Effects of PHELA^®^ Extract on *S. aureus* Biofilm

*S. aureus* was cultured on MHB and the concentration was adjusted to 0.5 McFarland standards. The biofilms were grown on coverslips using 12 well plates in the presence or absence of standard or test materials. The cleaned coverslips were immersed in piranha solution (Conc. H_2_SO_4_ and 30% H_2_O_2_ in a ratio of 3:1) for 15 min and washed with sterile MilliQ water and dried in the vacuum desiccators. After 16 h of incubation at 37 °C, the coverslips were taken out and dried for 12 h and put in desiccators [25,26]. AFM was utilized to study the microbial systems including structure, topographical landscape and behavior of biofilms. The samples were analyzed using AFM (di INNOVA, Bruker) run by NanoDrive v8 real-time control & NanoScope Analysis software in semi-contact mode at the speed of 1 Hz in tapping mode [27]. The surface and roughness of AFM images were analyzed using the image acquisition software [28,29]. The AFM data were collected at 1 × 1 μm scan area. The statistical parameters like Root Mean Square (Sq), Reduced peak height (Spk), Reduced valley depth (Svk), Roughness Average (Sa), Surface skewness (Ssk), Coefficient of kurtosis (Ska) Surface Area Ratio (Sdr), % Maximum height (Sz) values were measured using WSxM 5.0 software [30].

## 3. Statistical Analysis

All the experiments were performed in triplicate and the values were presented in mean ± SEM using GraphPad InStat Version 5.0 (GraphPad Software, Inc., San Diego, CA, USA).

## 4. Results

The UPLC-MS/MS analysis revealed the presence of 7 compounds from 6 different chemical classes like Phenolic acid or diterpene, flavonoids with corresponding glycosides or glycoside, anthraquinone derivatives, reducing disaccharide in both ionization mode. The amount of emodin present in the extract was determined by RP-HPLC analysis. The in-vitro cytotoxicity assay indicates the extract having a CC_50_ value > 1 mg/mL and the CYP450 assay data showed lesser interaction with 3A4 and 2D6 isozymes. The extract was found to be most active against *S. aureus* and a concentration-dependent decrease in *S. aureus* biofilm formation was observed. AFM analysis showed reduction in height, average roughness of biofilm.

### 4.1. UPLC-MS Analysis of PHELA^®^ Extract

**Figure 1:**
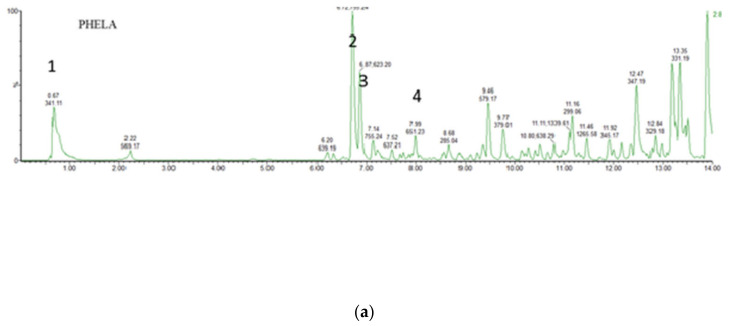

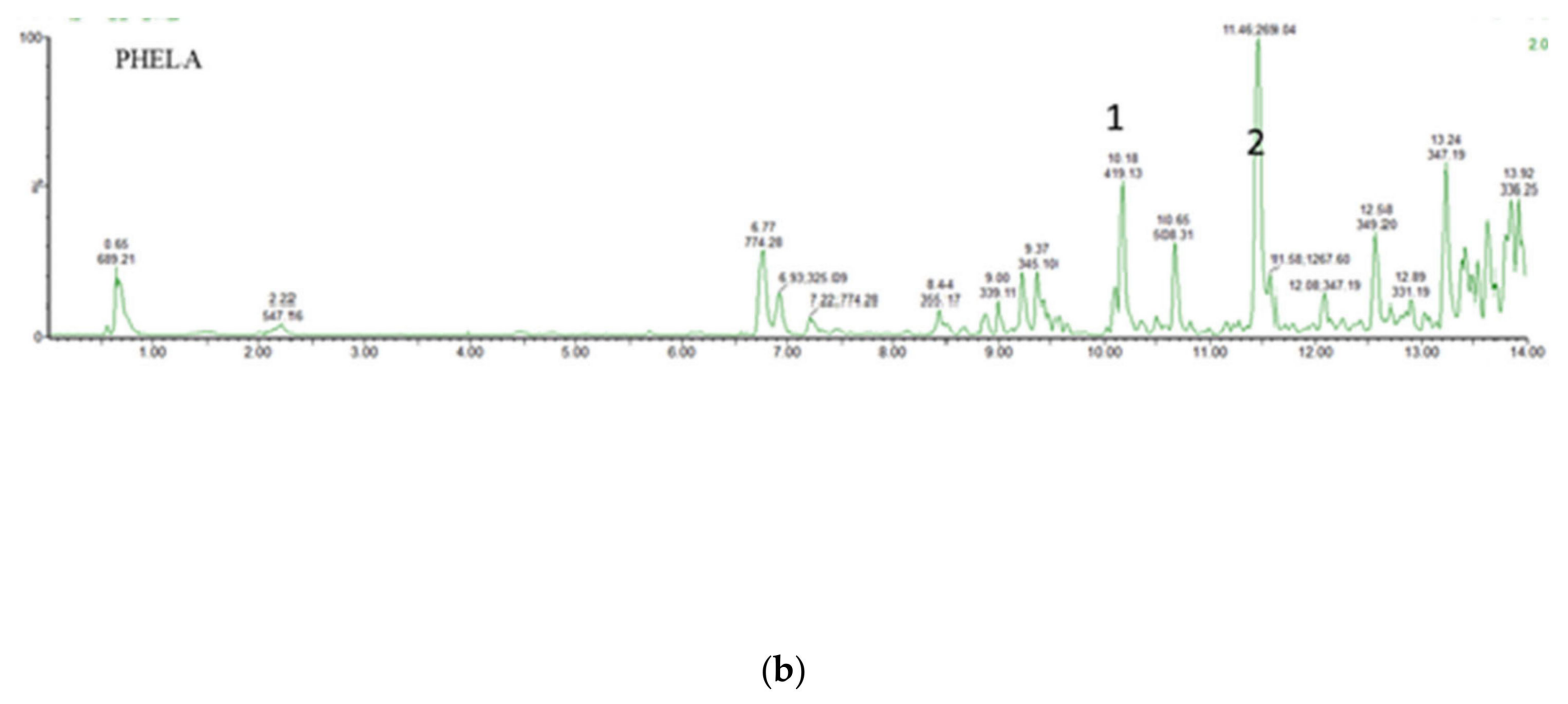
UPLC-QTOF-MS/MS total ion chromatogram for PHELA^®^ extract in negative ionization mode (**a**) and negative ionization mode (**b**).

Tentative identifications of compounds present in the hydro-alcohol extract of PHELA^®^ was carried out using LC-ESI-QTOF/MS in both positive and negative ionization modes by interpretation of their UV absorbance, molecular weight, and characteristic MS/MS fragment ions. A total of 7 compounds from 6 different chemical classes like Phenolic acid or diterpene, flavonoids with corresponding glycosides or glycoside, anthraquinone derivatives, reducing disaccharide were characterized and better fragments were collected in negative ion mode (Figure 1). A flavonoids glycoside along with a reducing disaccharide namely 6-0-α-d-glucopyranosyl-d-fructose and Quercetin-3-*O*-alpha-l-rhamnopyranosyl(1-2)-beta-d-glucopyranoside-7-*O*-alpha-l-rhamnopyranoside were detected at Rt 0.6 and 6.7 min showing molecular ion [M-H]^−^ at *m*/*z* values at 341.1089 and 755.2406 [31,32,33]. A chalcone derivative of bioflavonoid was identified to be Hesperidin methyl chalcone, showing [M-H]^−^ at *m*/*z* value of 623.1974 at 6.8 min [34]. Martynoside, a phenolic acid at 7.9 min produces molecular ion [M-H]^−^ at *m*/*z* 651.2285 [35]. Another compound at Rt 13.4 showed molecular ion [M-H]^−^ at *m*/*z* 331.1906 and was identified to be Carnosol [36]. In positive ionization mode, a flavonoid glycoside identified as Isoliquiritin at Rt 10.18 min showed [M+H]^+^ ion at *m*/*z* 419.1349 [37]. An anthraquinone class compound detected at Rt 11.4 showing [M+H]^+^ ion at *m*/*z* at 269.0448 and identified to be emodin. Table 1 shows the theoretical and acquired *m*/*z* values for individual compounds with error ppm ± 5 under library identification scores >80.

### 4.2. RP-HPLC Analysis of PHELA^®^ Extract

**Figure 2:**
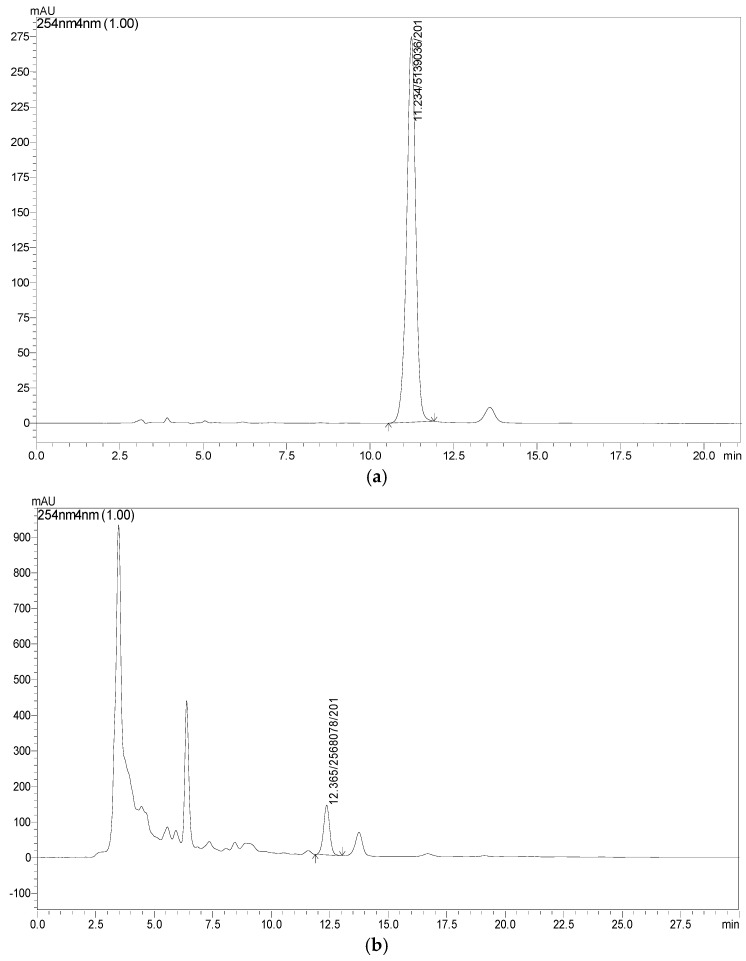
RP-HPLC chromatograms for standard emodin (**a**) and PHELA^®^ extract (**b**).

The RP-HPLC analysis data reveals the presence of emodin at an amount of 1.64% (*w*/*w*) using external standard calibration technique. The *r*^2^ value was 0.997 under the isocratic elution using the optimized mobile phase methanol and water (80:20) with Rt of 11.14 min (Figure 2).

### 4.3. Safety Assessment of PHELA^®^ Extract

The results obtained from in-vitro cytotoxicity assay shows that the extract has a CC_50_ value of 1250.45 ± 1.24 µg/mL where doxorubicin showed much more toxic against HepG2 cells with CC_50_ value of 5.25 ± 0.24 μg/mL at 24 h. The results indicate that the test material is least toxic in HepG2 cell line which justifies its safety aspects.

**Figure 3:**
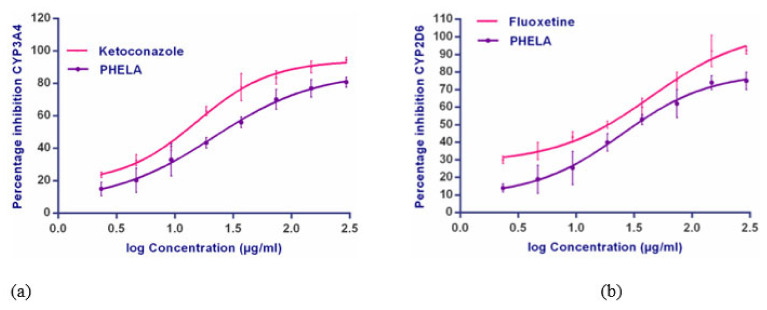
CYP3A4 (**a**) and CYP2D6 (**b**) inhibitory effect of PHELA^®^ extract and standard inhibitors.

The interaction potential of the PHELA^®^ formulation with different cytochromeP450 isozymes was performed using CYP450 isozymes inhibition assay to understand the safety aspect. Respective positive controls for each isozyme were used to confirm the assay precision. IC_50_ value of the extract and standards against CYP3A4 and CYP2D6 isozymes are represented in Figure 3. The IC_50_ values were found to be 0.2317 ± 2.14 and 0.2817 ± 3.65mg/mL against CYP3A4 and CYP2D6 for the extract. Results indicated that the PHELA^®^ extracts showed the least interaction potential against both the CYP-isozymes compared to standard inhibitors. The IC_50_ values for the standards were 0.0643 ± 1.12 and 0.0739 ± 1.69 mg/mL respectively (Table 2).

### 4.4. Antibacterial and Anti-Biofilm Activity of PHELA^®^ Extract

#### Determination of MIC and MBC

The antibacterial potency was evaluated using a 96 well microtitre plate broth dilution method against two strains of Gram-positive (*S. aureus* and *B. substilis*) and Gram-negative (*E. coli* and *P. aeruginosa*) bacteria. Streptomycin and ampicillin were used as positive control against Gram-positive and Gram-negative microorganisms, respectively. The MIC and MBC values were given in Table 3. From the bacterial susceptibility assay, PHELA^®^ extract was found to be active against both gram-positive and gram-negative bacteria in a dose-dependent manner. The PHELA^®^ extract showed the highest MIC value of 0.3125 ± 0.019 mg/mL against *S. aureus*.

### 4.5. Growth Curve Analysis

**Figure 4:**
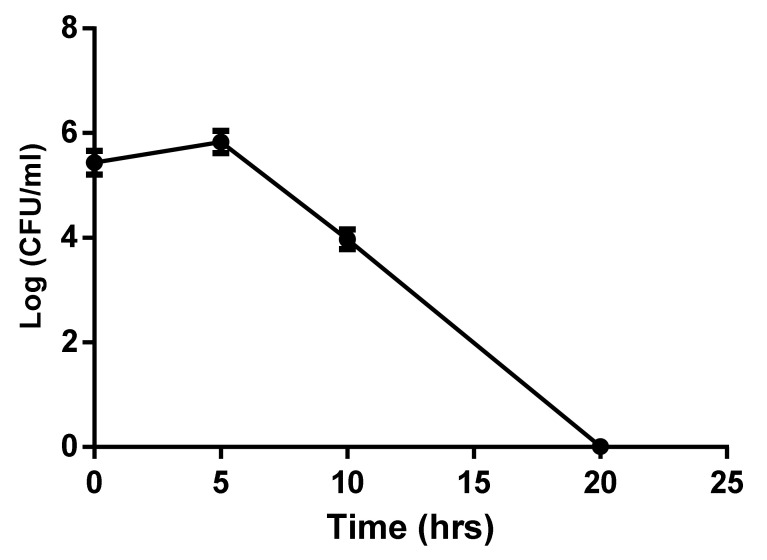
Growth curve of S. aureus against PHELA^®^ extract.

Growth curve experiments demonstrated that PHELA^®^ extract inhibited the growth of gram-positive microorganisms. Count been done in multiplication of dilution factor although almost no colony was found after 20 h treatment set (Figure 4).

### 4.6. Effect of PHELA^®^ on Formation of Biofilm

**Figure 5:**
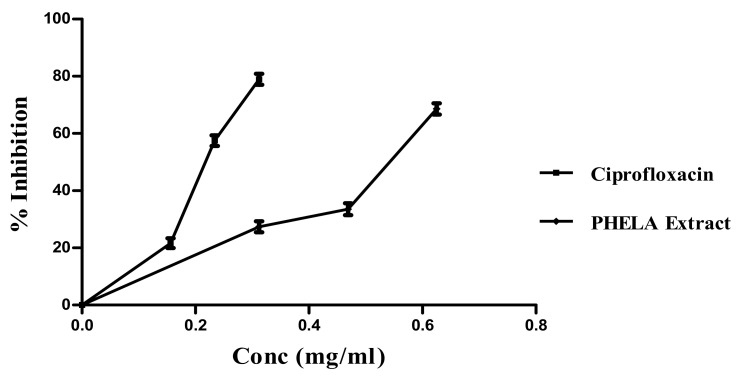
*S. aureus* biofilm inhibition of PHELA^®^ extract and ciprofloxacin.

A comparable concentration-dependent decrease in biofilm formation was observed by PHELA^®^ extract and Ciprofloxacin treatment. The extract produced 27.38 ± 1.57, 33.55 ± 1.68 and 68.59 ± 1.60% (0.312, 0.469 and 0.625 mg/mL) reduction in the biofilm produced by *S. aureus* where in case of ciprofloxacin the standard antimicrobial agent produced 21.67 ± 1.37, 57.53 ± 1.53 and 78.92 ± 1.57% (0.156, 0.234 and 0.312 mg/mL) inhibition of biofilm formation (Figure 5).

**Figure 6:**
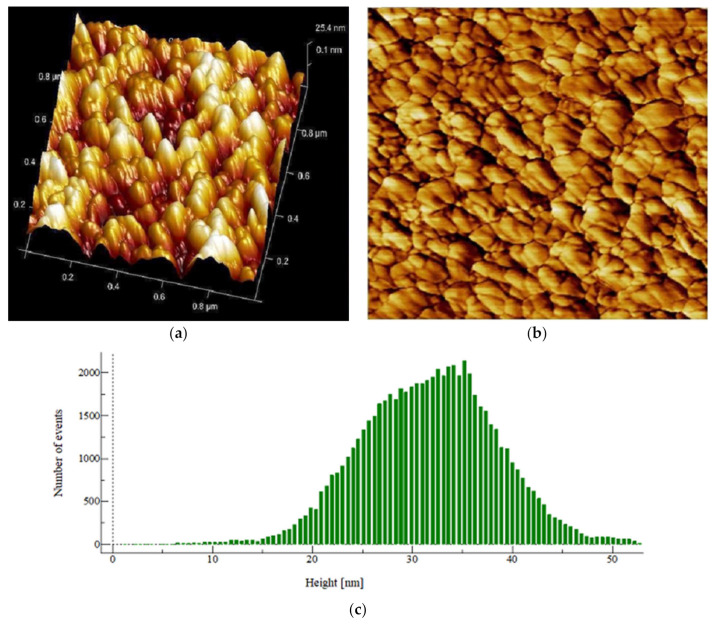
AFM image of *S. aureus* biofilm surface (untreated) formed on the polycarbonate membrane filter taken at the 1 × 1 μm scan area in the tapping mode. (**a**) Height topography 3D, (**b**) The structure and complete surface coverage of the biofilm, and (**c**) Height distribution pattern.

**Figure 7:**
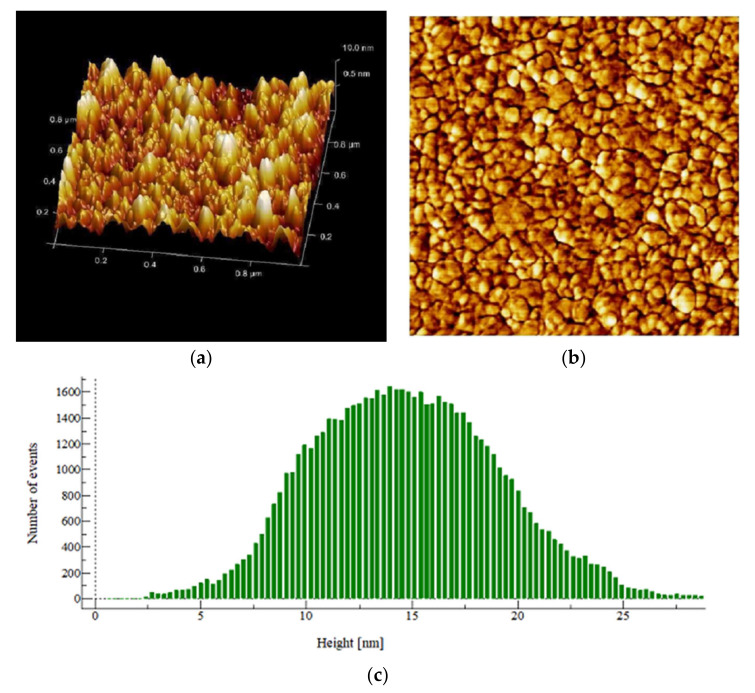
AFM image of *S. aureus* biofilm surface (treated with standard antimicrobial agent) formed on the polycarbonate membrane filter taken at the 1 × 1 μm scan area in the tapping mode. (**a**) Height topography 3D and (**b**) The structure and complete surface coverage of the biofilm, and (**c**) Height distribution pattern.

**Figure 8:**
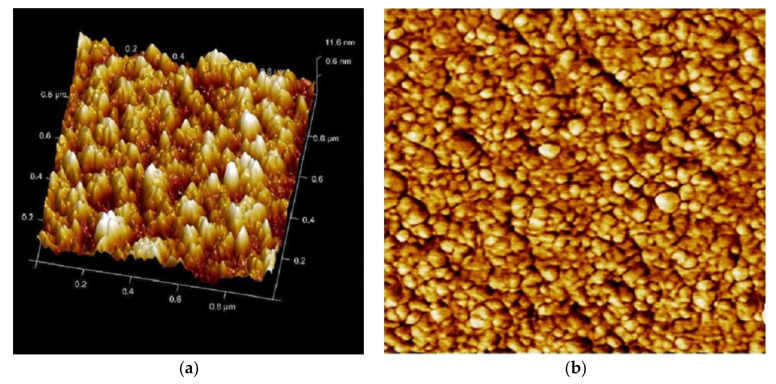

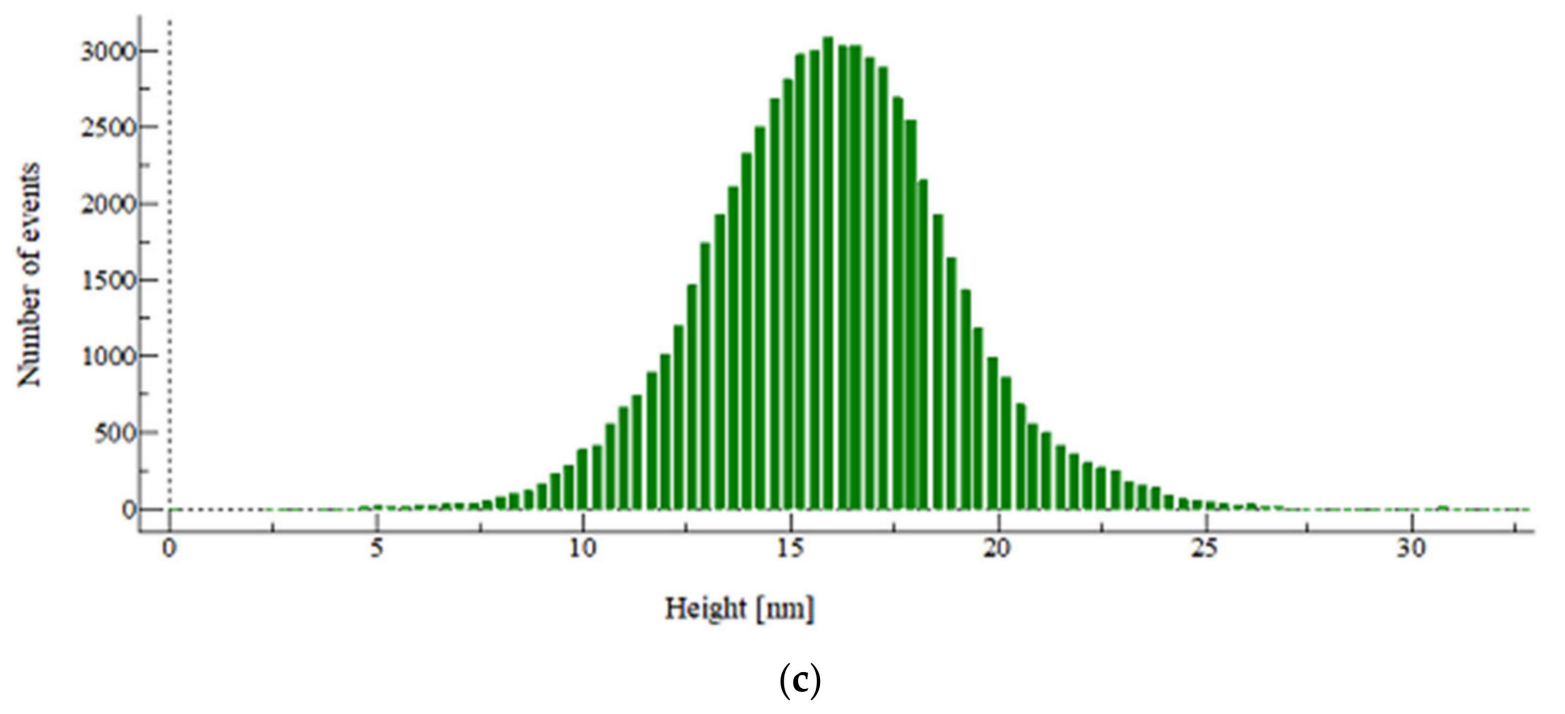
AFM image of *S. aureus* biofilm surface (treated with PHELA^®^ extract) formed on the polycarbonate membrane filter taken at the 1 × 1 μm scan area in the tapping mode. (**a**) Height topography in three dimensions, (**b**) The structure and complete surface coverage of the biofilm, and (**c**) Height distribution pattern.

There is a great utility of AFM for studying single-cell bacteria. It allows the measurement of the mechanical properties of biofilms with high spatial resolution in terms of rms, skewness, kurtosis and Gaussian distribution. The images of control showed an increase in statistical parameters (Sq, Spk, Svk, Sa, Ssk, Ska, Sdr, Sz, etc.) as compared to the PHELA^®^ and standard treated biofilm as calculated (Figure 6, Figure 7 and Figure 8). The most representative as the images obtained from the scans contain information about all the statistical parameters, and this information provided better visualization of the surface topology, three-dimensional micrographs (Table 4) [30].

## 5. Discussion

A biofilm is an evolving form of microbes that depend on the supra-cellular association that develops from the formation of the EPS matrix. Prevention of microbial cell attachment to surfaces is a better approach to inhibiting biofilm growth. The use of herbal medicine was found to be useful for decreasing microbial colony formation on surfaces which can afterwards turn into infections and become a major concern for immune-compromised patients [38,39,40]. Pretreatment of the surface with plant extracts produces an unfavorable film that promotes detachment, thereby reducing the surface adhesion. Although plant-derived antibacterial compounds generally demonstrate a lower degree of antimicrobial activity, the present study showed a dose-dependent reduction in biofilm formation by *S. aureus*, which remains a common causative organism associated with both nosocomial and community-acquired infections and wounds.

Antimicrobial activity of plant extracts is attributed to the presence of numerous bioactive secondary metabolites which are important components for exerting antimicrobial activity [41]. Many studies showed natural product-derived secondary metabolites with poor antimicrobial properties may exhibit synergy with different antimicrobial agents. Qualitative or quantitative characterization of herbal extract has enormous implications for validating its quality and the safety of the optimal concentrations of bio-active constituent present therein [42]. The data regarding the UPLC-ESI-QTOF/MS analysis shows the presence of seven metabolites in the extract from different phytochemical classes. The flavonoid derivative, Hesperidin methyl chalcone has anti-inflammation, skin wound healing, antimicrobial properties [43]. Martynoside was reported to be the most active compound from Boscia albitrunca, having potent antibacterial property against B. subtilis and K. pneumoniae [44]. Carnosol from *Salvia officinalis* was reported to potentiate the antimicrobial property of ethidium bromide, erythromycin and tetracycline [36]. In positive ionization mode, two compounds were identified to be present in the extract. Isoliquiritin, a flavonoid glycoside was characterized as is one of the major flavonoid glycoside extracted from Glycyrrhiza uralensis Fisch which helped to improve the antibacterial effect of meropenem, on NDM-1-positive Enterobacteria [37]. Another compound namely Emodin was found to be principal component in *P. cucumerina* possessing anti-biofilm and anti-virulence activities [45]. A study by Yan et al., showed emodin significantly decreased S. aureus biofilm growth in a dose-dependent manner by interrupting the release of extracellular DNA and inhibited expression of the biofilm-related genes by real-time RT-PCR [46]. The data regarding the RP-HPLC analysis shows the presence of emodin. These tentatively identified phytocompounds may contribute to the antibacterial and anti-biofilm property based on the available research works on individual phytocompounds. The statistical parameters calculated from AFM data showed a significant reduction in biofilm formation by PHELA^®^ extract as compared to the standard drug ciprofloxacin. AFM provide a unique insight into the control of microbial populations within clinical and industrial environments. The PHELA^®^ extract showed a significant reduction in statistical parameters as compared to the standard treated group [47]. The growth curve analysis data shows that the MBC concentration of PHELA^®^ extract inhibited the growth at different time intervals at different dilutions and almost no colony was found after 20 h treatment set.

The traditional poly herbal formulations which have been used for several hundred years by the traditional practitioners are thought to be safe for their therapeutic efficacy. However, the interaction of these herbal components with drug metabolizing enzymes may alter the pharmacokinetic profile leads to clinically significant drug–drug or herb–drug interaction [48].

The interaction potential of the PHELA^®^ formulation with different cytochromeP450 isozymes was examined using the CYP450 isozymes inhibition assay to understand the safety aspect. This assay was based on the ability of herb or herbal formulation to vie with the different fluorogenic substrate for different CYP isoforms. High throughput screening (HTS) for fluorimetric CYP inhibition is the most rapid and cost-effective approach to test the inhibition potential. The probe substrates used in these assays are derivatives of coumarin, which after dealkylation by CYPs generates fluorescent products. Respective positive controls for each isozyme were used to confirm the assay precision. Results indicated that the PHELA^®^ extracts showed the least interaction potential against both the CYP-isozymes compared to standard inhibitors.

CYP3A4 mostly expressed in the human liver and intestine. It is possibly the most significant drug-metabolizing enzyme in humans which covers up to 60% of the total hepatic cytochrome P450 content [49]. Over 60% of drug candidates presently in use are bio-transformed by this drug metabolizing enzyme. This CYP isoform shows significant interaction with macrolide antibiotics, anti-arrhythmics drugs, benzodiazepine, immunomodulators, antiretroviral, prokinetic agents, calcium channel blockers, HMG CoA reductase etc. [50,51]. Besides these, it is also involved in the metabolism of testosterone, progesterone and androstenedione [52,53]. The CYP2D6 subclass is mainly expressed in duodenum and brain and comprises about 2% of total hepatic cytochrome P450 content. It involves biotransformation of over 70 different drug candidates through oxidation. Different therapeutic classes of drugs, like β-adrenergic blockers, antiarrhythmics, antipsychotics, antidepressants, and narcotic analgesics are the substrates for this isozyme [54,55,56].

It should be noted that simultaneous co-administration of potentially interacting herbs/herbal formulation with drugs cases altered pharmacokinetic profile resulting in unwanted toxicity. The data from the CYP450 isozyme inhibitory assay showed that the PHELA^®^ formulation having higher IC_50_ values as compared to the standard inhibitor and thus will not be causing any harmful adverse reaction upon co-administration with the above-mentioned therapeutic classes of drugs.

## 6. Conclusions

New approaches should be applied for the control of biofilms because of the increased resistance of bacterial biofilms to different antimicrobial agents. In the present investigation, evaluation of therapeutic efficacy of PHELA^®^ formulation as an anti-infective through translational of traditional knowledge and its ability to prevent biofilm formation has been evaluated along with standardization by RP-HPLC. The standardized extract showed a dose-dependent reduction in biofilm formation by *S. aureus*. The least cytotoxic effect on HepG2 cell line and the cytochromeP450 isozyme inhibitory assay confirms its safety upon co-administration with CYP450 3A4 and 2D6 substrates drug candidates. It may be concluded that the PHELA^®^ extract may serve as a safe anti-infective agent against certain gram-positive bacteria might be due to its varied secondary plant constituents.

The present findings suggest that the PHELA^®^ formulation may provide a safe and effective alternative to treat infectious diseases caused by *S. aureus*, particularly through inhibition of biofilm formation. The identified phytocompounds may contribute to the antibacterial and anti-biofilm property.

## Figures and Tables

**Table 1:** Possible compounds tentatively identified in PHELA^®^ hydro-alcohol extracts using UPLC-ESI-QTOF-MS correspond to peaks as depicted in Figure 1 in both negative modes followed by positive ionization mode.

Rt (min)	Compound Name	Chemical Formula	Acquired [M-H]^−^ *m*/*z* Ratio	Theoretical [M-H]^−^ *m*/*z* Ratio	Calculated Accurate Mass (Da)	Mass Error (ppm)	References
0.6	6-0-α-D-glucopyranosyl-D-fructose	C_12_H_22_O_11_	341.1089	341.1084	342.1162	1.5	[32]
6.7	Quercetin-3-*O*-alpha-l-rhamnopyranosyl(1-2)-beta-D-glucopyranoside-7-*O*-alpha-l-rhamnopyranoside	C_34_H_44_O_19_	755.2406	755.2399	756.2476	0.9	[31,33]
6.8	Hesperidin methyl chalcone	C_29_H_36_O_15_	623.1974	623.1976	624.2054	−0.3	[34]
7.9	Martynoside	C_31_H_40_O_15_	651.2285	651.2289	652.2366	−0.6	[35]
13.4	Carnosol	C20H26O4	331.1906	331.1909	330.1831	−0.9	[36]
**Rt(min)**	**Compound name**	**Chemical formula**	**Acquired** **[M+H]^+^ *m*/*z***	**Theoretical [M+H]^+^ *m*/*z***	**Calculated accurate mass (Da)**	**Mass error (ppm)**	**References**
10.18	Isoliquiritin	C_21_H_22_O_9_	419.1349	419.1342	418.1263	1.7	[37]
11.4	Emodin	C_15_H_9_O_5_	269.0448	269.0450	268.0371	−0.7	[38]

**Table 2:** IC_50_ values (µg/mL) of PHELA^®^ formulation and individual plant extracts against CYP3A4 and CYP2D6 isozyme by the PHELA^®^ formulation and individual plant extracts.

Name of the Plant	CYP3A4 (mg/mL) ± SEM	CYP2D6 (mg/mL) ± SEM
PHELA^®^ formulation	0.2317 ± 2.14	0.2817 ± 3.65
Positive control	Ketoconazole	Fluoxetine
	0.0643 ± 1.12	0.0739 ± 1.69

**Table 3:** MIC and MBC values for PHELA^®^ extract.

Gram-Positive Bacteria	MIC (mg/mL)	MBC (mg/mL)
*S. aureus*	0.3125 ± 0.019	<1.00
*B. substilis*	0.356 ± 0.007	>1.00
**Gram-negative bacteria**		
*P. auregenosa*	0.625 ± 0.017	>1.25
*E. coli*	0.512 ± 0.011	>1.5

MIC: Minimum inhibitory concentration; MBC: Minimum bactericidal concentration.

**Table 4:** Statistical parameters for AFM analysis.

Statistical Parameters	Control	Standard	PHELA^®^ Extract
Root Mean Square (Sq)	9.165 nm	4.344 nm	3.523 nm
Reduced peak height (Spk)	36 nm	14.12 nm	17.51 nm
Reduced valley depth (Svk)	28.56 nm	14.75 nm	10.42 nm
Roughness Average (Sa)	7.30 nm	3.52 nm	2.81 nm
Surface skewness (Ssk)	0.19	0.14	0.4
Coefficient of kurtosis (Ska)	0.07	0.19	0.27
Surface Area Ratio (Sdr) %	5.98	1.75	1.90
Maximum height (Sz)	64.56 nm	28.87 nm	27.93 nm

## Data Availability

Not applicable.

## Abbreviations

EPS	Extracellular polymeric substances
MHB	Muller Hinton Broth
ATCC	American Type Culture Collection
PHAE	Hydro-alcoholic extract of PHELA^®^
RP-HPLC	Reverse Phase- High Performance Liquid Chromatography
PDA	Photodiode Array
CYP	Cytochrome
MIC	Minimum Inhibitory Concentration
MBC	Minimum Bactericidal Concentration
AFM	Atomic Force Microscopy
Sq	Root Mean Square
Spk	Reduced peak height
Svk	Reduced valley depth
Sa	Roughness Average
Ssk	Surface skewness
Ska	Coefficient of kurtosis
Sdr	Surface Area Ratio
Sz	% Maximum height
r2	correlation coefficient
Rt	Retention time
